# Le traitement conservateur médical de la fistule œsotracheale

**DOI:** 10.11604/pamj.2018.31.13.15491

**Published:** 2018-09-04

**Authors:** Fatogoma Issa Koné, Kadidiatou Singaré, Siaka Soumaoro, Naouma Cissé, N’Faly Konaté, Kassim Diarra, Yaya Dembélé, Samba Karim Timbo, Mohamed Amadou Keïta

**Affiliations:** 1 Service ORL et Chirurgie Cervico-Faciale, Centre Hospitalo-Universitaire Gabriel Touré, Bamako, Mali

**Keywords:** Fistule œsotrachéale, corps étranger, tomodensitométrie, TOGD, traitement médical, œso-tracheal fistula, foreign body, CT scan, EGD transit, medical treatment

## Abstract

Les fistules œsotrachéales représentent une entité pathologique caractérisée par la présence d'une communication anormale entre l'arbre trachéal et l'œsophage. A travers un cas clinique de fistule œsotrachéale suite à l'ingestion d'un corps étranger, nous abordions l'aspect étiopathogénique, évaluer notre principe thérapeutique et faire une revue de la littérature. Il s'agit d'un patient de 15ans admis pour dysphagie évoluant depuis deux mois suite à l'ingestion d'un corps étranger à type de morceau d'os. L'œsophagoscopie sous anesthésie générale a permis l'extraction du corps étranger. Le Transit Oeso-gastro-duodénal (TOGD) en post-opératoire a objectivé l'orifice fistulaire siégeant au niveau de C7. L'absence de l'orifice fistulaire a été notée à J30 post-endoscopie au TOGD de contrôle. Le traitement médical est une alternative thérapeutique de la fistule œsotrachéale et doit être basé sur des critères bien définis.

## Abstract (en)

œso-tracheal fistulas are a pathological condition characterized by an abnormal communication between the tracheal tree and the esophagus. We here report a clinical case of œso-tracheal fistula due to the ingestion of a foreign body in order to highlight its etiopathogenesis, to evaluate our therapeutic approach and to do a literature review. The study involved a 15 year old patient admitted with dysphagia evolving over the last two months following the ingestion of a foreign body (a piece of bone). œsophagoscopy under general anesthesia allowed extraction of the foreign body. Post-operative EGD transit objectified fistular orifice at the C7 level. The absence of fistular orifice was recorded 30 days after endoscopy during his follow-up EGD transit. Medical treatment is a therapeutic alternative for œso-tracheal fistula and must be based on well-defined criteria.

## Introduction

Les fistules œso-trachéales représentent une entité pathologique caractérisée par la présence d'une communication anormale entre l'arbre trachéale et le tube digestif à travers l'œsophage [1]. L'étiologie de la fistule est source de controverse en présence d'un corps étranger acéré: il est difficile de différencier l'origine iatrogénique de la fistule au cours des manœuvres d'extraction du corps et de la fistule due au corps lui-même [2]. Les corps étrangers sont responsables de 15-20% des perforations œsophagiennes [2]. Son spectre clinique est spécifique et se résume à une toux spontanée, s'accentue au cours de l'alimentation et la douleur cervicale [2]. La diversité thérapeutique donne le choix aux chirurgiens de procéder à un traitement médical conservateur, le traitement mini-invasif voire l'œsophagectomie [3]. La place du traitement médical n'étant pas mise en avant, cependant il est fonction du délai diagnostique, de la clinique, du terrain, de la possibilité de surveillance clinique et radiologique et une équipe rodée à la prise en charge médicochirurgicale [2-4]. Nous rapportons un cas de fistule œsotrachéale dû au corps étranger œsophagien acéré traité par un traitement médical conservateur. A travers ce cas nous abordions l'aspect étiopathogénique, évaluer notre principe thérapeutique et faire une revue de la littérature.

## Patient et observation

**Figure 1:**
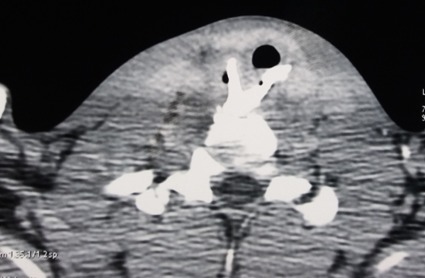
Tomodensitométrie pharyngolaryngée en coupe axiale mettant en évidence le corps étranger affleurant la lumière trachéale

Patient de 17 ans admis pour dysphagie évoluant depuis deux mois (2 mois). La dysphagie est apparue suite à l'ingestion d'un corps étranger à type de morceau de viande. Elle était apyrétique et d'évolution progressive. Elle s'intéressait aux liquides aussi bien qu'aux solides. L'adjonction d'une douleur antéro-cervicale, d'une hypersialorrhée et une toux productive, a été notée. La toux était spontanée et s'accentuait au cours de l'alimentation. Aucune notion de dysphonie, de dyspnée ni d'emphysème cervical n'a été notée. Une notion de massage traditionnel cervical à base de beurre de karité et un traitement médical non spécifié n'ont engendré aucun succès. Une pathologie psychiatrique non typée a été rapportée par les parents, aucune traçabilité n'a été relevée. L'état général était conservé avec les plis de dénutrition, une température à 37,6°C, une tension artérielle évaluée à 100/60 mm Hg, une pulsation artérielle radiale à 80 par minute et la fréquence respiratoire a été de 17 cycles par minute. Une diminution du murmure vésiculaire dans les deux champs pulmonaires avec des râles crépitants à l'examen pleuropulmonaire. Une induration douloureuse antéro-cervicale a été notée. Le reste de l'examen était sans particularité. Le patient était porteur d'une anémie microcytaire normochrome. La tomodensitométrie pharyngolaryngée (Figure 1) a conclu à des opacités oblongues hypo-pharyngées étendues au médiastin antérieur évoquant un corps étranger. Le corps étranger affleurait la lumière trachéale. Cet aspect tomodensitométrique fait suspecter une fistule œsotrachéale.

**Figure 2:**
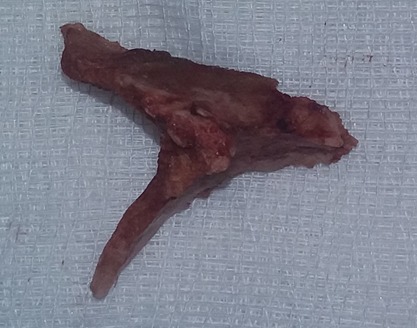
Corps étranger en forme de ‘V’

A L'œsophagoscopie le corps étranger était enclavé et entouré de granulome inflammatoire saignant au contact. Il était situé à 15 cm de l'arcade dentaire supérieure. Il s'agissait d'un morceau d'os en forme de ‘V’ (Figure 2), transversale à la lumière œsophagienne. L'extraction a été laborieuse et a ramené un morceau d'os mesurant environ 1cm. La muqueuse œsophagienne était inflammatoire, saignante et l'orifice fistulaire n'a pas été objectivé au contrôle endoscopique. La mise en place une sonde nasogastrique CH16 a été réalisée. Les suites opératoires ont été marquées par la constatation d'une fistule œso-trachéale à la hauteur de C7 au transit œsogastro-duodénale (TOGD) à J1 post opératoire. L'abstention thérapeutique chirurgicale a été de règle. Le traitement médical a été instauré. Il s'agit de: 1) ceftriaxone 1 gramme toutes les 12 heures pendant 3 semaines en intraveineuse directe(IVD); 2) paracétamol 1 gramme toutes les 6 heures en IVD pendant deux jours; 3) Lanzoprazol 30mg par jour pendant 1 mois; 4) la sonde nasogastrique d'alimentation à demeure. Le contrôle du trajet fistuleux par le TOGD a été établi en trois temps: à J7, J14, J30 en post-opératoire. Au décours des contrôles nous avons noté une diminution progressive du trajet fistuleux jusqu'à sa disparition à J30. Le retrait de la sonde nasogastrique et la reprise alimentaire par voie orale ont été établis. Avec un recul de trois mois aucun signe n'a été noté.

## Discussion

Les fistules œsotrachéales révèlent d'une diversité étiologique: iatrogénique, spontanée, traumatique, corps étranger, tumorale, chirurgicale [3]. L'ingestion de corps étranger est plus fréquente chez l'enfant que chez l'adulte [2].Les corps étrangers sont fréquents parmi les causes des fistules œsotrachéales [2]. Dans notre cas il s'agissait d'un corps étranger à type de morceau d'os. La nature migratoire, l'ancienneté du corps, la position acérée du corps et l'impaction prolongée du corps sur la muqueuse œsophagienne pouvant être responsable d'une inflammation locale, ont été rapportés comme facteurs pouvant engendrer une fistule [4]. A ces facteurs s'ajoute la faiblesse de la partie postérieure membraneuse de la trachée [5]. La nature pointue, ancienne, acérée a constitué les principaux facteurs pouvant engendrer une fistule œsotrachéale chez notre patient. Il est difficile d'imputer la cause de la fistule au corps étranger ou à la manœuvre endoscopique [2]. Ces facteurs pouvant tous engendrer la fistule œsotrachéale. Les endoscopies diagnostiques avec un fibroscope souple et rigide se compliquent respectivement dans 0,03% et 0,11% des cas de perforation [3]. La fistule a été suspectée dans notre cas avant la manœuvre endoscopique basée sur les caractéristiques de la toux et le résultat de la tomodensitométrie. A ces éléments de suspiçion s'ajoutent les signes de complications pulmonaires [2,6]. L'endoscopie et les examens radiologiques constituent une étape clé du diagnostic. L'endoscopie est un outil qui permet de visualiser la fistule. Elle montre ses limites devant la fistule de petite taille, cachée par les plis longitudinaux de la muqueuse [1-4]. Dans notre cas la fistule n'a pas été visualisée due à la présence des granulomes inflammatoires saignants autour de la zone d'impaction du corps étranger. La suspiçion diagnostique a été faite à la tomodensitométrie qui a double intérêt: la visualisation du corps étranger et la détection des signes de complications [2,3]. Aucune complication n'a été recensée chez le patient. Le TOGD est l'examen capital dans le diagnostic des fistules œsotrachéales. Il permet de déterminer le siège de l'orifice fistulaire [2,3]. Le TOGD a conforté la suspiçion diagnostique de la tomodensitométrie en préopératoire, en mettant en évidence la fistule à la hauteur de C7. La suspiçion diagnostique de la tomodensitométrie et la persistance de la toux en post-opératoire, ont été sa principale indication.

Les alternatives thérapeutiques sont variables, de caractère invasif croissant, et vont du traitement médical jusqu'à l'œsophagectomie, en passant par le traitement endoscopique interventionnel. Entre le traitement médical et la chirurgie ouverte tendent actuellement à se positionner des options mini-invasives comme l'endoscopie interventionnelle associée à des gestes de drainage chirurgicaux ou radiologiques; qui n'est pas encore validées [3,4,7,8]. Le traitement médical conservateur a été indiqué dans notre cas devant un état général conservé, l'absence de fièvre, des signes pulmonaires moins marqués et un état local de la muqueuse œsophagienne non infecté. Ces critères ont été rapportés dans l'étude de S. Kallel sur 573 patients colligés suite à une endoscopie pour corps étranger [4]. Il a prouvé l'intérêt du traitement médical chez 80% des patients porteurs de fistule [4]. L'utilisation d'antibiothérapie à large spectre de longue durée est admise comme dans notre cas. Le traitement anti reflux, l'alimentation à travers la sonde nasogastrique, ont servi d'aide dans la cicatrisation de la fistule chez notre patient comme dans le cas de S Kallel [4]. Le délai de guérison dans notre cas a été constaté à J30 au TOGD. Ce délai de guérison va de cinq jours et pouvant atteindre jusqu'à trois mois chez les patients traités par la méthode conservatrice [4].

## Conclusion

Le traitement médical d'une fistule œso-trachéale est une stratégie thérapeutique non invasive permettant d'obtenir une fermeture spontanée de l'orifice fistulaire. Néanmoins elle doit obéir à des critères bien codifiés.

## Conflits d’intérêts

Les auteurs ne déclarent aucun conflit d'intérêts.
